# FACTORS ASSOCIATED WITH FOOD NEOPHOBIA IN CHILDREN: SYSTEMATIC REVIEW

**DOI:** 10.1590/1984-0462/2021/39/2020089

**Published:** 2020-11-06

**Authors:** Thamara de Oliveira Torres, Daiene Rosa Gomes, Mússio Pirajá Mattos

**Affiliations:** a Universidade Federal do Oeste da Bahia, Barreiras, BA, Brazil.

**Keywords:** Food neophobia, Feeding behavior, Food preferences, Children, Systematic review, Neofobia alimentar, Comportamento alimentar, Preferências alimentares, Crianças, Revisão sistemática

## Abstract

**Objective:**

To identify the factors associated with food neophobia in children through a systematic review.

**Data sources:**

This research was based on the recommendations of the Preferred Reporting Items for Systematic Reviews and Meta-Analyses. The research was carried out in the PubMed, Science Direct, and Scientific Electronic Library Online databases, with the combination of health descriptors in English and Portuguese: (“Food Neophobia” OR “Feeding Behavior” OR “Food Preferences” OR “Food Selectivity”) AND Child, from 2000 to 2019. Studies that evaluated factors associated with food neophobia in children were included. The quality of the studies was assessed using the Effective Public Health Practice Project: Quality Assessment Tool for Quantitative Studies (QATQS).

**Data synthesis:**

19 studies were included in the systematic review. The prevalence of food neophobia ranged from 12.8 to 100%. The studies used three different scales to measure the level of food neophobia. The main factors associated with food neophobia were: parental influence on children’s eating habits, children’s innate preference for sweet and savory flavors, influence of the sensory aspect of the food, parents’ pressure for the child to eat, parents’ lack of encouragement and/or affection at mealtime, childhood anxiety, and diets with low variety and low nutritional quality.

**Conclusions:**

The factors associated with food neophobia permeate several areas of the child’s life, thus, interprofessional follow-up becomes essential in the intervention process.

## RESUMO

**Objetivo:**

Identificar os fatores associados à neofobia alimentar em crianças por meio de uma revisão sistemática.

**Fontes de dados:**

Esta pesquisa foi baseada nas recomendações do Preferred Reporting Items for Systematic Reviews and Meta-Analyses. A busca foi realizada nas bases de dados PubMed, ScienceDirect e Scientific Electronic Library Online (SciELO), com a conjugação dos descritores em saúde em português e inglês: (“*Food Neophobia*” OR “*Feeding Behavior*” OR “*Food Preferences*” OR “*Food Selectivity*”) AND *Child*, no período de 2000 a 2019. Foram incluídos os estudos que avaliaram os fatores associados à neofobia alimentar em crianças. A qualidade dos estudos foi mensurada por meio da ferramenta Effective Public Health Practice Project: Quality Assessment Tool for Quantitative Studies (QATQS).

**Síntese dos dados:**

Dezenove trabalhos foram incluídos na revisão sistemática. A prevalência da neofobia alimentar variou de 12,8 a 100%. Os estudos utilizaram três diferentes escalas a fim de medir o nível de neofobia alimentar. Os principais fatores associados a esse quadro foram: influência parental nos hábitos alimentares da criança, preferência inata das crianças por sabores doces e salgados, influência do aspecto sensorial do alimento, pressão dos pais para a criança comer, falta de encorajamento e/ou afetividade dos pais no momento das refeições, ansiedade na infância, dietas pouco variadas e com baixa qualidade nutricional.

**Conclusões:**

Os fatores associados à neofobia alimentar permeiam diversos âmbitos da vida da criança, assim, o acompanhamento interprofissional torna-se essencial no processo de intervenção.

## INTRODUCTION

Food neophobia is characterized by a reluctance to consume or an unwillingness to try unknown foods.^1^ This behavior, from an evolutionary perspective, can minimize risks of eating foods harmful to health; however, this aversion causes food monotony, which can result in nutritional deficiencies.^2^ ^,^ ^3^ Food should not be solely seen as a basic need, but also as a source of pleasure, socialization, cultural transmission, and a factor of great importance for health.^4^ These aspects influence the formation of eating habits, which mostly originate in childhood, and the family has a paramount role in learning how to feed the child.^5^

The neophobic behavior mainly occurs in the age group of two to five years, a significant period for the formation of eating habits.^6^ Due to the limited knowledge of this behavior, many parents do not identify it in their children, which reinforces the possibility that the prevalence of neophobia is even greater than the data reported in the literature.^5^ The lack of identification of neophobia is worrisome, considering that the foods that most drive it are of high nutritional value.^7^

The development of food neophobia is associated with several factors such as individual, biological, psychological, economic, anthropological, and sociocultural factors.^8^ Knowledge of such elements allows developing an adequate approach to face neophobia, considering that eating behavior may be differently influenced. For intervention to take place, it is essential to study this condition associated with other variables, especially the eating habits of those who most strongly influence the children’s food preferences.^9^

The lack of diversity in food caused by food neophobia restricts the intake of nutrients necessary to maintain the body homeostasis. When this restriction is severe and/or lasts for a long time, it tends to affect various systems of the human body, such as the nervous system, affecting the child’s cognitive and motor abilities.^10^

Considering the factors associated with food neophobia in children and its impact on their development, it is necessary to carry out studies on this issue in order to enable the dissemination of knowledge of neophobia and, consequently, its prevention, early identification, and appropriate intervention. It is worth considering that the difficult identification of neophobia causes this behavior to last long enough to severely affect the child’s development and health. Therefore, the present study aims to identify factors associated with food neophobia in children through a systematic review.

## METHOD

This is a systematic review study, based on the standards of the Preferred Reporting Items for Systematic Reviews and Meta-Analyses (PRISMA),^11^ on studies that evaluated the factors associated with food neophobia in children. In this review, the concept of food neophobia was adopted as the tendency to reject new or unknown foods.^1^ However, due to the conceptual confusion still present in the literature, there was need to include other descriptors in the search for articles.

To do so, an electronic investigation of articles indexed in the PubMed, ScienceDirect, and Scientific Electronic Library Online (SciELO) databases was carried out, with the combination of the following descriptors in English and Portuguese languages (DeSC/MeSH): (“Food Neophobia” OR “Feeding Behavior” OR “Food Preferences” OR “Food Selectivity”) AND Child and (“*Neofobia Alimentar*” OR “*Comportamento Alimentar*” OR “*Preferências Alimentares*” OR “*Seletividade Alimentar*”) AND *Criança*. Studies published from January 2000 to December 2019 were considered.

Articles that analyzed factors associated with food neophobia in children, published in Portuguese and in English, were included. Reviews, theses, dissertations, editorials, and studies that did not correlate with the used descriptors were excluded. Studies available from the databases were selected and analyzed by two independent reviewers (TOT and DRG), using forms that comprised the eligibility criteria, including the title, the abstract and, finally, the full article. Disagreements between the two reviewers were resolved in consultation with a third reviewer (MPM).

Relevant information of the selected articles was systematized in a Word spreadsheet containing the following data: authors, year of publication, study locations, study types, sample, quality score, prevalence of food neophobia in children, food neophobia scale, level of neophobia, source environment of the sample, and associated factors. The study location was described according to the country and city of performance. As for the temporal aspect, articles were presented according to the year of publication. The sample of each study was characterized by the number of participants.

The methodological quality of the selected articles was assessed by using the scale Effective Public Health Practice Project: Quality Assessment Tool for Quantitative Studies (QATQS) (https://merst.ca/wp-content/uploads/2018/02/quality-assessment-tool_2010.pdf). With this tool, publications were analyzed according to five components (classified as strong, moderate, or weak): selection bias, study design, confounders, data collection method, and type of analysis employed. Subsequently, the studies were classified as follows: (1) strong, for studies that did not present components classified as weak; (2) moderate, for studies that presented only one weak component; (3) weak, for studies that presented two or more components with the same classification.

## RESULTS

**Figure 1:**
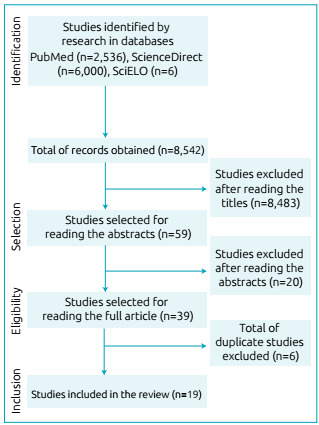
Flowchart of the search strategy and results from the databases.

The search strategies are shown in Figure 1. A total of 8,542 articles were identified in the databases. The excluded studies consisted in review articles, theses, dissertations, and editorials, in addition to duplicates; those that did not address food neophobia in childhood were also excluded. Thus, 19 articles were selected for analysis in the systematic review.^2^ ^,^ ^12^ ^,^ ^13^ ^,^ ^14^ ^,^ ^15^ ^,^ ^16^ ^,^ ^17^ ^,^ ^18^ ^,^ ^19^ ^,^ ^20^ ^,^ ^21^ ^,^ ^22^ ^,^ ^23^ ^,^ ^24^ ^,^ ^25^ ^,^ ^26^ ^,^ ^27^ ^,^ ^28^ ^,^ ^29^

The main characteristics of the selected studies are presented in Table 1. When classifying them according to type of study, the exclusive presence of cross-sectional research was noteworthy. The selected studies were carried out in 10 different countries, with the United States of America consisting in the country with the most publications (five articles),^2^ ^,^ ^12^ ^,^ ^13^ ^,^ ^14^ ^,^ ^15^ followed by Australia (four articles).^11^ ^,^ ^16^ ^,^ ^17^ ^,^ ^18^ The selected studies were conducted in the years 2000,^2^ 2003,^12^ 2006,^19^ 2008,^16^ 2010,^20^ 2012,^14^ ^,^ ^17^ 2014,^21^ ^,^ ^22^ 2015,^18^ ^,^ ^23^ 2016,^13^ ^,^ ^24^ 2017,^15^ ^,^ ^25^ ^,^ ^26^ 2018^27^ ^,^ ^28^ and 2019.^29^ Concerning the language, the 19 articles were written in English. The sample size of the studies ranged from 70 to 560 children of diverse origin in criteria such as: school environment,^2^ ^,^ ^15^ ^,^ ^16^ ^,^ ^19^ ^,^ ^20^ ^,^ ^22^ ^,^ ^23^ ^,^ ^25^ ^,^ ^26^ ^,^ ^27^ ^,^ ^28^ ^,^ ^29^ home environment,^12^ ^,^ ^13^ ^,^ ^14^ hospital environment,^24^ and pediatric outpatient clinic.^17^ ^,^ ^18^ ^,^ ^21^

**Table 1:** Characteristics of the studies selected in the systematic review of food neophobia in children.

Author/year	Study location/type	Sample	Quality score
Falciglia et al. (2000)^2^	United States of America - Cincinnati/Cross-sectional	70 children	Weak
Galloway et al. (2003)^12^	United States of America - Pennsylvania/Cross-sectional	192 children (7 years)	Weak
Cooke et al. (2006)^19^	United Kingdom - London/Cross-sectional	109 children (average age of 9 years)	Moderate
Russell and Worsley (2008)^16^	Australia - Burwood/Cross-sectional	371 children (2 to 5 years)	Moderate
Mustonen and Tuorila (2010)^20^	Finland - Helsinki/Cross-sectional	164 children (8 to 11 years)	Moderate
Howard et al. (2012)^17^	Australia - Brisbane and Adelaide/Cross-sectional	277 children	Moderate
Tan and Holub (2012)^14^	United States of America - Dallas/Cross-sectional	85 children (3 to 12 years)	Moderate
Cassells et al. (2014)^21^	Australia - Adelaide/Cross-sectional	244 children	Moderate
Laureati et al. (2014)^22^	Italy - Milan/Cross-sectional	560 children	Moderate
Maratos and Staples (2015)^23^	England - Derby/Cross-sectional	70 children (8 to 11 years)	Weak
Perry et al. (2015)^18^	Australia - Brisbane/Cross-sectional	330 children (2 years)	Moderate
Kaar et al. (2016)^13^	United States of America - Aurora/Cross-sectional	210 children (3 to 5 years)	Moderate
Moding and Stifter (2016)^24^	United States of America - Pennsylvania/Cross-sectional	115 children	Moderate
Kozioł-Kozakowska et al. (2018)^25^	Poland - Krakow/Cross-sectional	325 children (2 to 7 years)	Weak
Helland et al. (2017)^26^	Norway - Kristiansand/Cross-sectional	505 children (mean age of 2 years)	Weak
Maiz and Balluerka (2018)^15^	Spain - San Sebastián/Cross-sectional	464 children	Moderate
Rioux et al. (2018)^27^	France - Lyon and Paris/Cross-sectional	109 children (3 to 4 years)	Moderate
Kähkönen et al. (2018)^28^	Finland - Helsinki/Cross-sectional	130 children (3 to 5 years)	Moderate
Kutbi et al. (2019)^29^	Saudi Arabia - Jeddah/Cross-sectional	216 children (3 to 7 years)	Moderate

**Figure 2:**
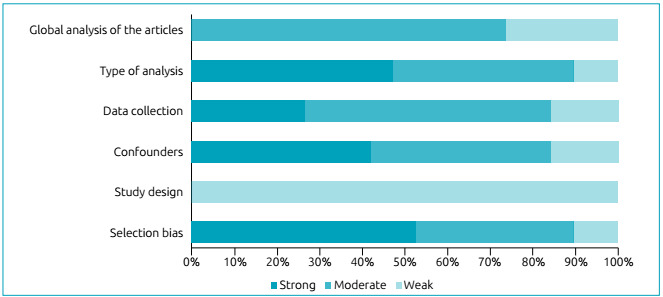
Summarization of the global methodological rigor of studies selected for the systematic review of factors associated with food neophobia in children.

In the evaluation of methodological rigor, according to the QATQS criteria, 14 (74%) articles were classified as moderate^13^ ^,^ ^14^ ^,^ ^15^ ^,^ ^16^ ^,^ ^17^ ^,^ ^18^ ^,^ ^19^ ^,^ ^20^ ^,^ ^21^ ^,^ ^22^ ^,^ ^23^ ^,^ ^24^ ^,^ ^27^ ^,^ ^28^ ^,^ ^29^ and 5 (26%), as weak.^2^ ^,^ ^12^ ^,^ ^23^ ^,^ ^25^ ^,^ ^26^ The study design was the item that contributed to moderately classify the quality of the study; on the other hand, precision in data collection and confounders were the items that most contributed to the weak methodological rigor (Figure 2).

The prevalence and level of food neophobia in childhood are highlighted in Table 2. The prevalence of food neophobia was present in 10 (53%) studies and varied between 12.8%^18^ and 100%.^29^ To identify neophobia, some scales were used, such as Pliner and Hobden’s,^13^ ^,^ ^16^ ^,^ ^17^ ^,^ ^20^ ^,^ ^21^ ^,^ ^22^ ^,^ ^23^ ^,^ ^25^ ^,^ ^29^ Pliner’s^14^ ^,^ ^19^ ^,^ ^24^ ^,^ ^26^ ^,^ ^27^ or Maiz, Balluerka, and Maganto’s.^15^ Based on these instruments, the level of neophobia was estimated, obtaining an average score consistent with the moderate ^13^ ^,^ ^14^ ^,^ ^16^ ^,^ ^17^ ^,^ ^20^ ^,^ ^24^ ^,^ ^25^ ^,^ ^26^ ^,^ ^27^ and high^15^ ^,^ ^19^ ^,^ ^21^ ^,^ ^22^ ^,^ ^23^ ^,^ ^29^ classification for neophobia.

**Table 2:** Prevalence and level of food neophobia in childhood.

Author/year	Prevalence (%)	Neophobia scale	Prevailing level of neophobia
Falciglia et al. (2000)^2^	32.8	-	-
Galloway et al. (2003)^12^	33	-	-
Cooke et al. (2006)^19^	47.7	Child Food Neophobia Scale - Pliner, 1994	High
Russell and Worsley (2008)^16^	-	Child Food Neophobia Scale - Pliner and Hobden, 1992	Moderate
Mustonen and Tuorila (2010)^20^	-	Food Neophobia Scale - Pliner and Hobden, 1992	Moderate
Howard et al. (2012)^17^	-	Food Neophobia Scale - Pliner and Hobden, 1992	Moderate
Tan and Holub (2012)^14^	-	Child Food Neophobia Scale - Pliner, 1994	Moderate
Cassells et al. (2014)^21^	13	Food Neophobia Scale - Pliner and Hobden, 1992	High
Laureati et al. (2014)^22^	-	Food Neophobia Scale - Pliner and Hobden, 1992	High
Maratos and Staples (2015)^23^	27.1	Food Neophobia Scale - Pliner and Hobden, 1992	High
Perry et al. (2015)^18^	12.8	-	-
Kaar et al. (2016)^13^	-	Food Neophobia Scale - Pliner and Hobden, 1992	Moderate
Moding and Stifter (2016)^24^	-	Child Food Neophobia Scale - Pliner, 1994	Moderate
Kozioł-Kozakowska et al. (2018)^25^	87.7	Food Neophobia Scale - Pliner and Hobden, 1992	Moderate
Helland et al. (2017)^26^	-	Child Food Neophobia Scale - Pliner, 1994	Moderate
Maiz and Balluerka (2018)^15^	28.7	Spanish Child Food Neophobia Scale - Maiz, Balluerka, and Maganto, 2016	High
Rioux et al. (2018)^27^	-	Child Food Neophobia Scale - Pliner, 1994	Moderate
Kähkönen et al. (2018)^28^	24.5	-	-
Kutbi et al. (2019)^29^	100	Food Neophobia Scale - Pliner and Hobden, 1992	High

As for factors associated with this condition in children, the following stand out: parental influence on eating habits,^17^ ^,^ ^23^ children’s innate preference for sweet and savory flavors,^18^ ^,^ ^19^ influence of the sensory aspect of foods,^20^ ^,^ ^23^ ^,^ ^27^ ^,^ ^29^ parents’ pressure for the child to eat,^13^ ^,^ ^21^ ^,^ ^24^ ^,^ ^29^ parents’ lack of encouragement and/or affection at mealtime,^22^ ^,^ ^24^ diets with low variety and low nutritional quality,^2^ ^,^ ^12^ ^,^ ^14^ ^,^ ^17^ ^,^ ^18^ ^,^ ^19^ ^,^ ^22^ ^,^ ^23^ ^,^ ^26^ childhood anxiety,^12^ ^,^ ^15^ little time to prepare the meals,^12^ mothers with food neophobia,^12^ limited availability of variety of foods,^14^ lack of exposure to new foods,^16^ preference for foods rich in fat and/or sugar,^16^ parents’ difficulty in interpreting signs of hunger and satiety,^21^child’s lack of autonomy in eating,^23^ negative reactions to new stimuli,^24^family residing in rural areas,^25^ and low education level of mothers^28^ (Table 3).

**Table 3:** Source environment of the sample and factors associated with food neophobia in children.

Author/year	Source environment of the sample	Associated factors
Falciglia et al (2000)^2^	School environment	Diet with low variety and quality.
Galloway et al. (2003)^12^	Home environment	Childhood anxiety; Mothers with food neophobia; Little time to prepare meals.
Cooke et al. (2006)^19^	School environment	Diet with low variety and quality; Children’s innate preference for sweet and savory flavors.
Russell and Worsley (2008)^16^	School environment	Diet with low variety and quality; Lack of exposure to new foods; Preference for fats and/or sugars.
Mustonen and Tuorila (2010)^20^	School environment	Diet with low variety and quality; Sensory aspect of the food.
Howard et al. (2012)^17^	Pediatric outpatient clinic	Diet with low variety and quality; Parental influence on eating habits.
Tan and Holub (2012)^14^	Home environment	Diet with low variety and quality; Limited availability of variety of foods; Preference for fats and/or sugars.
Cassells et al. (2014)^21^	Pediatric outpatient clinic	Mothers’ food beliefs; Parents’ pressure for children to eat; Parents’ difficulty in interpreting hunger/satiety.
Laureati et al. (2014)^22^	School environment	Diet with low variety and quality; Parents’ lack of encouragement
Maratos and Staples (2015)^23^	School environment	Diet with low variety and quality; Visual aspect of the food.
Perry et al. (2015)^18^	Pediatric outpatient clinic	Diet with low variety and quality; Children’s innate preference for sweet and savory flavors.
Kaar et al. (2016)^13^	Home environment	Parental influence on eating habits; Parents’ pressure for children to eat; Children’s lack of autonomy in eating.
Moding and Stifter (2016)^24^	Hospital environment	Negative reactions to new stimuli; Parents’ pressure for children to eat; Parents’ lack of encouragement and/or affection
Kozioł-Kozakowska et al. (2018)^25^	School environment	Diet with low variety and quality; Family residing in rural areas.
Helland et al. (2017)^26^	School environment	Diet with low variety; Sensory aspects of food.
Maiz and Balluerka (2018)^15^	School environment	Anxiety signs in childhood; Worse social, physical, and academic self-concept.
Rioux et al. (2018)^27^	School environment	Sensory aspect of the food; Low inductive reasoning.
Kähkönen et al. (2018)^28^	School environment	Diet with low variety and quality; Mothers’ low education level.
Kutbi et al. (2019)^29^	School environment	Parents’ pressure for children to eat; Sensory aspect of the food.

## DISCUSSION

Food neophobia is a behavior prevalent in childhood because it is a period of tactile, taste-related, and olfactory discoveries and when eating habits are formed. This prevalence is even greater when considering that, sometimes, neophobia is not identified.^5^ The causality that determines it has not yet been fully recognized. This phenomenon is determined by the interaction between several complex factors such as: biological, anthropological, economic, psychological, and/or sociocultural factors, which are shaped by the individual context.^8^

In this review, a high variability in the prevalence of food neophobia in children was observed. Such prevalence is determined by age, and its development is deemed greater from two to five years of age.^5^ ^,^ ^6^ During puberty and adulthood, the risk of developing this attitude toward food significantly decreases; however, in old age, it increases again, which is explained by the fact that neophobic behavior can protect the organism from possible intoxication due to old age.^27^ Studies performed by Kozioł-Kozakowska et al., ^25^ who analyzed the prevalence of food neophobia in the population of Polish preschool children, showed that this attitude is observed in 1 out of 10 children.

Some of the selected studies used the food neophobia scales developed by Pliner and Hobden,^13^ ^,^ ^16^ ^,^ ^17^ ^,^ ^20^ ^,^ ^21^ ^,^ ^22^ ^,^ ^23^ ^,^ ^25^ ^,^ ^29^ Pliner^14^ ^,^ ^19^ ^,^ ^24^ ^,^ ^26^ ^,^ ^27^, or by Maiz, Balluerka, and Maganto^15^ to measure the level of this behavior in the sample. This heterogeneity of instruments reinforces the findings of Damsbo-Svendsen,^30^ who pinpointed the diversity of tools used to measure food neophobia due to different strengths and weaknesses, considering that no instrument is sufficiently adequate to measure all involved aspects. The most used scale was that of Pliner and Hobden, composed of 10 items that seek to analyze the willingness to try new foods; however, its limitation is the fact that it does not include foods from different cultures.^31^ In addition, it is a scale formulated for the adult population, which can generate distorted results when applied to children.

Conversely, the Pliner scale is a version of the aforementioned scale, adapted for children aging 5 to 11 years and that comprises 34 foods. Parents report their children’s familiarity with these foods and their willingness to try them.^1^ Among its limitations, it does not include foods from different cultures either and is formulated for an age group higher than that found in several studies whose authors have used it. The third scale is aimed at children and adolescents, a Spanish version, culturally adapted from the Food Situation Questionnaire (FSQ), composed of 10 items also seeking to assess the willingness to try new foods.^32^ Taking into account the weaknesses and strengths of the aforementioned instruments, it is paramount to choose the one that best evaluates food neophobia in the target audience, in such a way not to generate inconsistent results.

The average score presented in the articles that used this tool indicated a higher occurrence of the moderate ^13^ ^,^ ^14^ ^,^ ^16^ ^,^ ^17^ ^,^ ^20^ ^,^ ^24^ ^,^ ^25^ ^,^ ^26^ ^,^ ^27^ and high^15^ ^,^ ^19^ ^,^ ^21^ ^,^ ^22^ ^,^ ^23^ ^,^ ^29^ levels of neophobia, which is a worrisome factor, because it demonstrates greater dietary restrictions as well as greater reluctance to new foods. High levels of food neophobia are associated with a lack of variety in food and a high intake of saturated fat, contributing to a diet with low nutritional quality.^2^ Among nutrients commonly restricted when facing this behavior, vitamin E, folate, calcium, zinc, and fibers stood out.^2^ It should be considered that such nutrients are essential for maintaining health, especially in childhood, which is a period of development in which nutritional deficiencies can lead to poor physical and intellectual development, impairment of the nervous and immune system, future occurrence of chronic non-communicable diseases, among other associated morbidities.^10^

Among the associated factors, the parental influence on eating habits stands out.^17^ ^,^ ^23^ This encompasses several other factors observed in this study, such as mothers who present neophobia to the same foods as their children,^12^ considering that their diets have low consumption of vegetables, an important food group for adequate nutrition and which is the target of neophobic behaviors.^12^ ^,^ ^16^ Studies corroborate that the mother’s high level of neophobia is correlated with the highest neophobia in children.^33^ ^,^ ^34^ ^,^ ^35^ ^,^ ^36^ Thus, the importance of parents in having adequate eating habits as a strategy to reduce food neophobia in childhood is reinforced.

The role of parents in forming adequate eating habits in children has been evidenced in the literature.^14^ ^,^ ^16^ ^,^ ^23^ ^,^ ^37^ ^,^ ^38^ ^,^ ^39^ Children’s eating behaviors are shaped by observation and imitation of the behavior and reactions of people around them.^38^ Children tend to follow their parents’ habits due to the affective bond, which includes taking an interest in the same foods consumed by them.^23^ The study conducted by Harper and Sanders showed that children were much more likely to try an unknown food when, at the same time, their mothers also ate the product and reacted with enthusiasm. This effect was stronger than when the parents just verbally encouraged the child to try the food.^39^ Thus, it is important for parents to be willing to include such foods in their habits, in such a way they arouse the child’s interest.

One of the parents’ explanation for the low supply of nutritionally rich foods was the rush to eat due to a busy routine,^12^ in such a way that they resort to foods that are easy to prepare and that are mostly of low nutritional value, in addition to being rich in sodium and fats. Furthermore, it is important to include the child when preparing the food. The analysis performed by van der Horst^40^ showed that the involvement of children in the preparation of meals can reduce the intensity of neophobic behaviors and contribute to the construction of positive experiences with food. Thus, the importance of availability, accessibility to varied and high-nutritional value foods, and the inclusion of children in the preparation of meals in the family environment is noteworthy.

In this environment, not only exposure to these foods is important, but also the way they are offered, considering that a factor strongly associated with food neophobia is the parent’s pressure for children to eat^13^ ^,^ ^21^ ^,^ ^24^ ^,^ ^29^ and the parents’ lack of encouragement and/or affection at mealtime.^22^ ^,^ ^24^ This pressure often results from the parents’ difficulty in interpreting the signs of hunger and satiety, associated with concerns about the low weight of their children.^21^ In addition, the absence of affective behavior during meals contributes to children associating this moment with displeasure, with a mere physiological need. Thus, the importance of establishing a good relationship between the child and the food is disregarded. This relationship will contribute to arouse interest in the flavors, textures, and sensations of the food. These are emotional aspects that have a great influence on the emergence of neophobic behaviors. Therefore, the environment during mealtime must promote the pleasure in eating.

Parents’ pressure for children to eat foods they do not like results in greater resistance to their consumption. Studies have confirmed that the more authoritative the parents are during the mealtime, the more often the child rejects the offered foods.^17^ ^,^ ^35^ ^,^ ^41^ This corroborates the conclusions of Rigal et al.^42^ , who found it difficult to feed children aged 20 to 36 months mainly as a result of authoritarian coercive practices on the part of their parents, who force the child to consume the rejected foods. Interestingly, the authors also pointed out that a permissive feeding style, in which the parents satisfy all the child’s wishes to avoid food conflicts, does not increase the child’s willingness to try unknown food. Thus, parents must assess their child’s subjectivities and choose the best path to succeed in introducing food.

The innate predilection for sweet and savory flavors and the aversion to bitter and acidic substances consisted in one of the factors associated with food neophobia. There is a low level of acceptance on the part of children for new food products whose predominant taste is bitterness or acidity. Such behavior may potentially contribute to shaping the neophobic behavior toward specific types of food, especially those with a distinctly bitter taste.^34^ ^,^ ^43^ ^,^ ^44^ Many analyses have shown that vegetables consist in the most frequently rejected food group due to hypersensitivity to bitter taste.^37^ ^,^ ^44^ ^,^ ^45^ ^,^ ^46^ Hence, the importance of introducing different flavors in the child’s diet stands out, in order to shape future food preferences.

Likewise, breastfeeding becomes extremely important, considering that during the consumption of breast milk the child has the possibility to taste many flavors, depending on the type of food chosen by the mother. This contributes to the greater acceptance of new foods when introducing foods to children, mainly foods that the mother regularly consumed during pregnancy and lactation.^47^ Children fed with milk formula tend to get used to the constant and specific taste of the mixture and, consequently, show less tolerance or even aversion during exposure to new foods.^37^ ^,^ ^43^ ^,^ ^44^ ^,^ ^48^ A study performed by Mennella et al.^49^ found that children whose mothers consumed carrot juice in the third trimester of pregnancy and/or during breastfeeding were more likely to eat carrot puree when compared with children whose mothers did not drink the juice. Such information highlights the importance of healthier food choices since pregnancy and lactation, in order to minimize neophobic behavior in childhood.

The child’s resistance to eat certain foods can also be conditioned by the late introduction of new products in the diet. The openness to taste unknown flavors is greater in babies aging up to 12 months and decreases with age.^37^ In addition, the continuous exposure of a new food after the child’s initial negative reaction may result in the need to eliminate that food from the daily diet.^47^ Researchers have shown that only 10 to 15 positive experiences are sufficient to result in the food product acceptance.^38^ ^,^ ^47^ ^,^ ^48^ Furthermore, the sensory perception of new foods, with varying appearance, consistency, and texture, can weaken the children’s reluctance to eat them later in life.^47^ ^,^ ^50^ Another common mistake in the introduction of food is the imposition of the parents’ food preferences, preventing children from knowing different foods and exercising their own food choices.^51^ These findings reinforce the importance of the necessary care in the child’s food introduction in order to reduce food neophobia.

Diets with low variety and low nutritional quality^2^ ^,^ ^12^ ^,^ ^14^ ^,^ ^17^ ^,^ ^18^ ^,^ ^19^ ^,^ ^22^ ^,^ ^23^ ^,^ ^26^ are characteristics strongly associated with food neophobia, since this behavior does not often restrict the amount of ingested food, but commonly affects the feeding quality. Children with this behavior have a restricted diet, mainly regarding nutrients required for the maintenance of health, which causes severe nutritional deficiencies and, consequently, contributes to the emergence of morbidities.

The low variety and nutritional quality affect several food groups, mainly vegetables, meats, and fruits,^16^ foods rich in nutrients and important allies for an adequate diet. Another factor that contributes to an unbalanced diet is the fact that neophobic behaviors to these foods are commonly associated with high consumption of fats and sugars.^2^ ^,^ ^16^ In addition, the children’s innate preference for sweet and savory flavors means that these foods are not the target of neophobia.^18^ ^.^ ^19^

Restricted diets are mainly linked to parental influence, considering that parents and other people in the child’s environment usually offer only what is part of the family’s eating habits and their food beliefs.^21^ Such conduct deprives children of trying new foods, often not giving them the necessary autonomy in their feeding.^13^ Thus, it is necessary for food to be available to children even if they are not part of the family’s eating habits, in such a way to stimulate their autonomy in choosing their food.

Another factor that affects the attitude toward food is childhood anxiety. Anxiety^12^ ^,^ ^15^ is a common emotional disorder in neophobic children, and is associated with the fact that the parents of these children are not used to encouraging them to actively participate in their meal,^13^ such as in choosing the food and preparing the meal, in addition to the frequent pressure to eat exerted by the parents.^13^ ^,^ ^21^ ^,^ ^24^ ^,^ ^29^ It has been demonstrated that when children are forced to eat foods they do not want to, they start feeling anxious and tense, and their distaste for the foods increases.

This contributes to the development of negative associations related to the consumption of meals, and leads to the exacerbation of neophobic behaviors.^37^ ^,^ ^41^ ^,^ ^52^ It was also noted that these children have difficulties in socializing in the school environment, which affects their social, physical, and academic self-concept.^15^ Therefore, making the moment of eating pleasurable, with affective behaviors, and inducing the child’s participation in the meals can minimize the occurrence of anxiety signs, considering that they consist in alternatives that provide safety and autonomy to the child.

Moreover, a positive correlation was found between food neophobia and negative reactions to new stimuli,^24^ ^,^ ^27^ even when they were not food stimuli. Children who had a higher level of neophobia feared novelties, thus avoiding objects and foods with unknown shapes, colors, or textures. Conducting studies for analyzing the presence of anxiety before and after the occurrence of food neophobia would be relevant to enable a better characterization of the association of anxiety with food neophobia in children.

The place of residence was also a factor associated with neophobic behavior in childhood. Children living in urban areas had a lower level of food neophobia compared with those living in rural areas.^25^ This characteristic may be related to the availability of food in these places, since the access to variety is usually difficult in rural areas^53^. Therefore, it must be considered that exposure and availability to new foods are essential aspects to avoid or intervene in food neophobia.

Another factor related to food neophobia in childhood was the parents’ low level of education.^28^ This relation is explained by the parents’ insufficient knowledge to distinguish which food is adequate for their children with regard to: nutritional composition, handling, preparation, and appropriate exposure to food throughout life. Considering that this knowledge plays an important role in attenuating the neophobic behavior,^16^ the parents’ low level of education may contribute to the greater supply of food of low nutritional quality and the lower supply of those with rich nutritional value, which may result in the display of neophobic behaviors to these foods.^28^ Thus, the importance of food and nutrition education to parents is highlighted, in order to inform them about adequate food for children as well as to help them identifying, avoiding, or intervening in food neophobia.

Food and nutrition education becomes an essential tool for enabling an active, playful, and interactive process, with encouragement of previous experiences to facilitate the voluntary adoption of eating habits or any behavior related to food that leads to health and welfare.^54^ Furthermore, its advantages are the easy application and low cost, since this practice can be developed individually or in the school environment, for example. There is also sensory-based food education, which has been promising in the intervention of neophobic responses.^27^ This type of activity stimulates curiosity about new shapes, colors, and textures and can contribute to the emotional support and encouragement required to the care of children with neophobic behaviors, when developed in a conducive and comforting environment.

In this sense, the early identification of food neophobia allows for adequate intervention, preventing further damages to the child’s health, considering that this long-term behavior can affect the physical, cognitive, and psychosocial development.^5^ ^,^ ^7^ ^,^ ^10^ The interprofessional approach is paramount in this process due to the complexity that permeates this condition. It is believed that interprofessional care,^55^ through interaction between different knowledge and professional practices, enables a collective conduct aiming at promoting healthy eating habits. From this perspective, the nutritional follow-up becomes essential to prevent, mitigate, or eradicate food neophobia, and this assistance is not only aimed at the child, but also at the family, considering that the family environment is one of the greatest influences in the occurrence of this behavior.^5^ Therefore, a care network is encouraged, in such a way to promote healthy, happy, creative, fun, affective, and pleasurable feeding habits and focused on the child’s healthcare needs.

It is noteworthy that this review and the studies included in it have certain limitations, such as the scarcity of Brazilian studies and samples from different sources, in addition to the great variability of scales to measure food neophobia, which makes data comparability impossible. This lack of standardization may have been responsible for the wide variation in the prevalence of food neophobia. Hence, an individualized perspective of each research is interesting, taking into account that the lack of uniformity of the neophobia scales can cause generalizations and misinterpretations.

In conclusion, food neophobia significantly determines the children’s eating habits. The prevalence found in the selected studies confirms that it is a behavior easily displayed in childhood, especially at higher levels, which are related to severe dietary restrictions and impacts on health. Factors associated with food neophobia mainly referred to the parental influence on eating habits. However, these factors comprised several areas of the child’s life, demonstrating the importance of interprofessional follow-up throughout the intervention process.

Accordingly, adopting practices related to food and nutrition education should also be encouraged to enable the deepening of the knowledge of human feeding habits, especially in childhood. Thus, based on the information found in this study, there is need to offer a varied diet, also including foods that are not part of the family eating habits. In order to avoid or intervene in food neophobia, it is necessary to give children autonomy in their feeding habits, to emotionally support them, in addition to motivating them to participate in the preparation of meals, making the moment pleasant and affectionate.
